# *Rickettsia mongolotimonae* Infection in South Africa

**DOI:** 10.3201/eid1001.020662

**Published:** 2004-01

**Authors:** Anne-Marié Pretorius, Richard J. Birtles

**Affiliations:** * University of the Free State, Bloemfontein, South Africa; † University of Liverpool, Liverpool, United Kingdom

**Keywords:** Rickettsia mongolotimonae, lymphangitis, rashless, international tourist attraction site, South Africa

## Abstract

We report the first laboratory-confirmed case of *Rickettsia mongolotimonae* infection in Africa. The patient sought treatment for an eschar on his toe; lymphangitis, severe headaches, and fever subsequently developed. After a regimen of doxycycline, symptoms rapidly resolved. *R. mongolotimonae* infection was diagnosed retrospectively by serologic tests and molecular-based detection of the organism in biopsy specimens of eschar material.

Rickettsioses are infections of emerging medical importance, particularly in southern Africa, where an increasing number of cases are being encountered among both residents and tourists (*1*). Three *Rickettsia* species have been associated with human disease in South Africa to date. *Rickettsia conorii* has long been recognized as the agent of Mediterranean spotted fever, and more recently, a newly recognized species, *R. africae*, has been identified as the agent of African tick-bite fever. In 2002, the first case report of a patient infected with *R. aeschlimannii* was published (*2*). In addition to these recognized pathogens, *Rickettsia* species, including *R*. *mongolotimonae*, have been detected in human-biting arthropods in Africa. This species (*3*) was first encountered in *Hyalomma asiaticum* ticks in Inner Mongolia in 1991 (*4*) but has subsequently been associated with human infections in southern France (*5*) and, perhaps of most relevance to this report, has been detected in *H. truncatum* ticks collected from cattle in Niger (*6*). This species of tick, which at least during its immature life stages parasitizes migratory birds, is widely distributed in many African countries, including South Africa (*7*).

## The Study

In September 2002, a 34-year-old (HIV-seronegative) construction worker, working near Ellisras in South Africa’s Northern Province, discovered a lesion on the inside of the second toe on his right foot (Figure); subsequently, severe headaches and high fever developed. He was examined at a local hospital and found to have lymphangitis extending pretibially from the lesion; as a result of his other symptoms, he was treated for blood poisoning with ceftriaxone sodium, 1,000 mg once daily. During the next 3 days, the lesion at the bite site (noted by the examining physician) remained very sore, and the patient’s right inguinal lymph node became enlarged and very painful. The patient then decided to return to his hometown and sought treatment from his general practitioner (on day 5 after discovery of the lesion). On examination, the lesion and lymphangitis were clearly visible on the patient’s toe, although cellulitis and edema were not observed. His inguinal lymph node had swollen to 3 cm in diameter, and he was still febrile (38.5°C). Blood samples were then obtained as well as a biopsy specimen from the lesion. A regimen of doxycycline, 100 mg per day orally, for 5 days was prescribed and 1 day’s dosage was administered. The next day, the patient was afebrile, and the lymphangitis had completely resolved.

**Figure:**
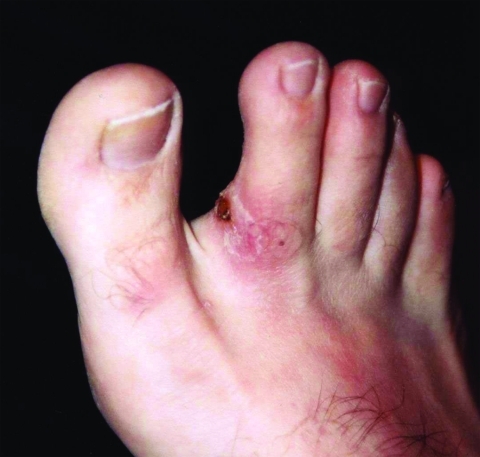
Eschar with lymphangitis.

In the laboratory, a Giemsa stain of a smear prepared from the patient’s blood showed activated lymphocytes. A complete blood count showed thrombocytosis (632 x 10^3^/μL), but all other hematologic parameters were within the normal range. Biochemical findings showed elevated levels of alanine transaminase (66 IU/L), blood urea nitrogen (7.2 g/L), and triglycerides (2.2 mmol/L); and decreased levels of chloride (96.3 mmol/L) and albumin (38g/L); all other tests yielded results within the normal range. Testing of the patient’s serum with the Weil-Felix test demonstrated an antibody titer (80) only against the OX2 *Proteus* antigen, giving presumptive evidence of a rickettsial infection. As a result, antirickettsial microimmunofluorescence testing was performed (*8*). The serum did not yield significant immunoglobulin (Ig) M titers against *R. conorii* or *R. africae* antigens, but IgG titers of 64 were found by using both antigens. DNA was extracted from the eschar biopsy specimen by using the QIAamp Tissue kit (QIAGEN GmbH, Hilden, Germany) according to the manufacturer's instructions. This DNA extract was used as template in a previously described polymerase chain reaction assay targeting a *Rickettsia* spp. *rOmpA* fragment (*9*). An amplification product was obtained from this extract but not from any concurrently processed control materials. The amplification product was purified; the nucleic acid sequences of both strands were then determined. The sequence obtained from these efforts was found to share >99% similarity with the corresponding *rOmpA* fragment of *R*. *mongolotimonae*.

## Conclusions

The combination of clinical and laboratory data yielded strong evidence that the case described here was an infection of *R. mongolotimonae*, the first reported in southern Africa. A single eschar is also typical of *R. conorii* infections, but these are characterized by rash (Mediterranean spotted fever), which the current patient did not have. Although *R. africae* infections manifest only rarely as a rash, they are typified by multiple eschars (*10*). This case description is also very similar to that relating to a French patient infected with *R. mongolotimonae*, who had lymphangitis and inguinal lymphadenopathy. The serologic findings indicate exposure to a spotted fever group rickettsia rather than to a specific species within this group, but the near identity of the *rOmp*A sequence obtained from the patient’s eschar to that of *R. mongolotimonae* provides a clear indication that this species, rather than other spotted fever group rickettsiae, was present at the site of the tick bite. Although no tick was found in association with the patient’s eschar, his infection may have been acquired from a *H. truncatum*, as this species is abundant in the region of the bushveld where the patient had been working and is known to feed on humans (*11*).

## References

[1] Raoult D, Fournier P-E, Fenollar F, Jensenius M, Prioe T, De Pina JJ, *Rickettsia africae*, a tick-borne pathogen of travellers to sub-saharan Africa. N Engl J Med. 2001;344:1504–10. 10.1056/NEJM200105173442003 11357153

[2] Pretorius A-M, Birtles RJ. *Rickettsia aeschlimannii*: a new pathogenic spotted fever group rickettsia, South Africa. Emerg Infect Dis. 2002;8:874. 12141981 10.3201/eid0808.020199 PMC2732514

[3] Raoult D, Brouqui P, Roux V. A new spotted-fever-group rickettsiosis. Lancet. 1996;348:412. 10.1016/S0140-6736(05)65037-4 8709763

[4] Yu X, Fan M, Xu G, Liu Q, Raoult D. Genotypic and antigenic identification of two new strains of spotted fever group rickettsiae isolated from China. J Clin Microbiol. 1993;31:83–8. 8093253 10.1128/jcm.31.1.83-88.1993 PMC262626

[5] Fournier P-E, Tissot-Dupont H, Gallais H, Raoult D. *Rickettsia mongolotimonae*: a rare pathogen in France. Emerg Infect Dis. 2000;6:290–2. 10827119 10.3201/eid0603.000309 PMC2640873

[6] Parola P, Inokuma H, Camicas J-L, Brouqui P, Raoult D. Detection and identification of spotted fever group rickettsiae and ehrlichiae in African ticks. Emerg Infect Dis. 2001;7:1014–7. 11747731 10.3201/eid0706.010616 PMC2631901

[7] Walker J. A review of the Ixodid ticks (Acari, Ixodidae) occurring in southern Africa. Onderstepoort J Vet Res. 1991;58:81–105. 1881661

[8] Teysseire N, Raoult D. Comparison of Western immunoblotting and microimmunofluorescence for diagnosis of Mediterranean spotted fever. J Clin Microbiol. 1992;30:455–60. 1537916 10.1128/jcm.30.2.455-460.1992 PMC265077

[9] Roux V, Fournier P-E, Raoult D. Differentiation of spotted fever group rickettsiae by sequencing and analysis of restriction fragment length polymorphism of PCR amplified DNA of the gene encoding the protein rOmpA. J Clin Microbiol. 1996;34:2058–65. 8862558 10.1128/jcm.34.9.2058-2065.1996 PMC229190

[10] Roux V, Raoult D. Rickettsioses as paradigms of new or emerging infectious diseases. Clin Microbiol Rev. 1997;10:694–719. 9336669 10.1128/cmr.10.4.694 PMC172941

[11] Horak IG, Fourie LJ, Heyne H, Walker JB, Needham GR. Ixodid ticks feeding on humans in South Africa: with notes on preferred hosts, geographic distribution, seasonal occurrence and transmission of pathogens. Exp Appl Acarol. 2002;27:113–36. 10.1023/A:1021587001198 12593517

